# Individualized Atrophy‐Based Prediction of Dementia Progression in Familial Frontotemporal Lobar Degeneration With Bayesian Linear Mixed‐Effects Modeling

**DOI:** 10.1002/ana.78167

**Published:** 2026-01-29

**Authors:** Shubir Dutt, Dana Leichter, Yann Cobigo, Amy Wolf, John Kornak, Annie Clark, Lucy L. Russell, Arabella Bouzigues, David M. Cash, Martina Bocchetta, Molly Olzinski, Brian Appleby, Ece Bayram, Barbara Borroni, Andrea Bozoki, Chris R. Butler, David Clark, Rhian S. Convery, R. Ryan Darby, Alexandre de Mendonça, Bradford Dickerson, Kimiko Domoto‐Reilly, Simon Ducharme, Eve Ferry‐Bolder, Elizabeth Finger, Phoebe H. Foster, Douglas R. Galasko, Daniela Galimberti, Alexander Gerhard, Nupur Ghoshal, Caroline Graff, Neill Graff‐Radford, Ian M. Grant, Chadwick M. Hales, Lawrence S. Honig, Ging‐Yuek Hsiung, Edward D. Huey, David Irwin, Lize C. Jiskoot, Walter Kremers, Justin Y. Kwan, Robert Laforce, Isabelle Le Ber, Gabriel C. Léger, Johannes Levin, Irene Litvan, Ian R. Mackenzie, Mario Masellis, Mario F. Mendez, Fermin Moreno, Chiadi Onyike, Markus Otto, Belen Pascual, Peter Pressman, Rosa Rademakers, Eliana Marisa Ramos, Aaron Ritter, Erik D. Roberson, James B. Rowe, Raquel Sanchez‐Valle, Isabel Santana, Harro Seelaar, Allison Snyder, Sandro Sorbi, Matthis Synofzik, Maria Carmela Tartaglia, Pietro Tiraboschi, John C. van Swieten, Marijne Vandebergh, Rik Vandenberghe, Hilary W. Heuer, Bruce L. Miller, William W. Seeley, Maria Luisa Gorno‐Tempini, Joel H. Kramer, Leah Forsberg, Kejal Kantarci, Bradley F. Boeve, Adam L. Boxer, Jonathan D. Rohrer, Howard J. Rosen, Adam M. Staffaroni, FTD Prevention Initiative (FPI) investigators

**Affiliations:** ^1^ Department of Neurology, Edward and Pearl Fein Memory and Aging Center University of California, San Francisco San Francisco CA; ^2^ Dementia Research Centre, UCL Queen Square Institute of Neurology University College London London UK; ^3^ Department of Neurology Case Western Reserve University Cleveland OH USA; ^4^ Department of Neurology University of Colorado Aurora CO USA; ^5^ Department of Clinical and Experimental Sciences University of Brescia Brescia Italy; ^6^ Molecular Markers Laboratory, Fatebenefratelli, IRCCS Istituto Centro San Giovanni di Dio Brescia Italy; ^7^ Department of Neurology University of North Carolina Chapel Hill NC; ^8^ Nuffield Department of Clinical Neurosciences, Medical Sciences Division University of Oxford Oxford UK; ^9^ Department of Brain Sciences Imperial College London London UK; ^10^ Department of Neurology Indiana University Indianapolis IN; ^11^ Department of Neurology Vanderbilt University Nashville TN; ^12^ University of Lisbon Lisbon Portugal; ^13^ Department of Neurology Massachusetts General Hospital and Harvard Medical School Boston MA; ^14^ Department of Neurology University of Washington Seattle WA; ^15^ Department of Psychiatry Douglas Mental Health University Institute, McGill University Montreal Quebec Canada; ^16^ Montreal Neurological Institute, McConnell Brain Imaging Centre, McGill University Montreal QC Canada; ^17^ Department of Clinical Neurological Sciences University of Western Ontario London Ontario Canada; ^18^ Department of Neurosciences University of California – San Diego La Jolla CA; ^19^ Fondazione Ca’ Granda, IRCCS Ospedale Policlinico Milan Italy; ^20^ Centro Dino Ferrari University of Milan Milan Italy; ^21^ Division of Psychology Communication and Human Neuroscience, Wolfson Molecular Imaging Centre University of Manchester Manchester UK; ^22^ Department of Nuclear Medicine, Center for Translational Neuro‐ and Behavioral Sciences University Medicine Essen Essen Germany; ^23^ Department of Geriatric Medicine, Klinikum Hochsauerland Arnsberg Germany; ^24^ Departments of Neurology and Psychiatry Washington University, School of Medicine, Washington University St. Louis MO; ^25^ Department of Neurobiology, Division of Neurogeriatrics Karolinska Institutet Solna Sweden; ^26^ Unit for Hereditary Dementias Karolinska University Hospital Solna Sweden; ^27^ Department of Neurology Mayo Clinic Jacksonville FL; ^28^ Department of Psychiatry and Behavioral Sciences Northwestern Feinberg School of Medicine Chicago IL; ^29^ Department of Neurology Emory University School of Medicine Atlanta GA; ^30^ Taub Institute for Research on Alzheimer's Disease and the Aging Brain, College of Physicians and Surgeons Columbia University New York NY; ^31^ Department of Neurology Columbia University New York NY; ^32^ Department of Psychiatry, College of Physicians and Surgeons Columbia University New York NY; ^33^ Division of Neurology University of British Columbia Vancouver British Columbia Canada; ^34^ Department of Neurology and Penn Frontotemporal Degeneration Center, Perelman School of Medicine University of Pennsylvania Philadelphia PA; ^35^ Department of Neurology, Erasmus Medical Centre Rotterdam The Netherlands; ^36^ Department of Quantitative Health Sciences, Mayo Clinic Rochester MN; ^37^ National Institute of Neurological Disorders and Stroke, National Institutes of Health Bethesda MD; ^38^ Département des Sciences Neurologiques, Clinique Interdisciplinaire de Mémoire Laval University Quebec City Quebec Canada; ^39^ Paris Brain Institute Sorbonne Université Paris France; ^40^ Département de Neurologie Hôpital Pitié‐Salpêtrière Paris France; ^41^ Department of Neurology Ludwig‐Maximilians Universität München Munich Germany; ^42^ German Center for Neurodegenerative Diseases (DZNE) Munich Germany; ^43^ Munich Cluster of Systems Neurology (SyNergy) Munich Germany; ^44^ Department of Pathology University of British Columbia Vancouver British Columbia Canada; ^45^ Sunnybrook Health Sciences Centre, Sunnybrook Research Institute University of Toronto Toronto Ontario Canada; ^46^ Department of Neurology University of California – Los Angeles Los Angeles CA; ^47^ Department of Neurology Cognitive Disorders Unit, Hospital Universitario Donostia San Sebastian Spain; ^48^ Neurosciences Area, Group of Neurodegenerative Diseases Biogipuzkoa Health Research Institute San Sebastian Spain; ^49^ Center for Biomedical Research in Neurodegenerative Disease Carlos III Health Institute Madrid Spain; ^50^ Department of Psychiatry and Behavioral Sciences Johns Hopkins University Baltimore MD; ^51^ Department of Neurology University of Ulm Ulm Germany; ^52^ Department of Neurology Houston Methodist Research Institute Houston TX; ^53^ VIB Center for Molecular Neurology, VIB Antwerp Belgium; ^54^ Department of Biomedical Sciences University of Antwerp Antwerp Belgium; ^55^ Department of Neuroscience Mayo Clinic Jacksonville FL; ^56^ Lou Ruvo Center for Brain Health, Cleveland Clinic Las Vegas NV; ^57^ Department of Neurology University of Alabama at Birmingham Birmingham AL; ^58^ Department of Clinical Neurosciences and Cambridge University Hospitals NHS Trust University of Cambridge Cambridge UK; ^59^ Department of Neurology, Alzheimer's Disease and Other Cognitive Disorders Unit University of Barcelona Barcelona Spain; ^60^ University Hospital of Coimbra Neurology Service University of Coimbra Coimbra Portugal; ^61^ Center for Neuroscience and Cell Biology University of Coimbra Coimbra Portugal; ^62^ Department of Neurofarba University of Florence Florence Italy; ^63^ IRCCS Fondazione Don Carlo Gnocchi Florence Italy; ^64^ Department of Neurodegenerative Diseases, Hertie‐Institute for Clinical Brain Research and Center of Neurology University of Tübingen Tübingen Germany; ^65^ Tanz Centre for Research in Neurodegenerative Diseases University of Toronto Toronto Ontario Canada; ^66^ Fondazione IRCCS Istituto Neurologico Carlo Besta Milan Italy; ^67^ Department of Neurosciences Laboratory for Cognitive Neurology, KU Leuven Leuven Belgium; ^68^ Neurology Service University Hospitals Leuven Leuven Belgium; ^69^ Leuven Brain Institute Leuven Belgium; ^70^ Department of Neurology Mayo Clinic Rochester MN; ^71^ Department of Radiology Mayo Clinic Rochester MN

## Abstract

**Objective:**

Age of symptom onset is highly variable in familial frontotemporal lobar degeneration (f‐FTLD). Accurate prediction of onset would inform clinical management and trial enrollment. Prior studies indicate that individualized maps of brain atrophy can predict conversion to dementia in f‐FTLD. We used a Bayesian linear mixed‐effect (BLME) prediction method for identifying accelerated brain volume loss to predict conversion to dementia.

**Methods:**

Participants included 234 asymptomatic or prodromal carriers of *C9orf72*, *GRN*, or *MAPT* mutations (including 21 dementia converters) with ≥3 longitudinal magnetic resonance imaging (MRI) T1‐weighted scans. The BLME models established individual voxel‐wise gray matter trajectories using the first 2 scans. Person‐specific clusters of accelerated volume loss were estimated in subsequent scans and tested as predictors of dementia conversion compared with other approaches in time‐varying Cox proportional hazard models covarying for age. Receiver‐operating characteristic (ROC) curves estimated utility of cluster volume in discriminating which participants converted to dementia within 24 months.

**Results:**

The BLME cluster volume predicted conversion to dementia in f‐FTLD mutation carriers overall and separately in *C9orf72*, *GRN*, and *MAPT*, with comparable hazard ratios observed for atrophy W‐maps and regional volumes. Within a 24‐month timeframe, BLME cluster volume discriminated dementia converters from non‐converters with larger areas under the curve (AUCs) than other approaches.

**Interpretation:**

Bayesian‐modeled individualized atrophy scores predict dementia progression among asymptomatic f‐FTLD mutation carriers and may have increased utility compared with other structural imaging methods when studying individuals over shorter timeframes that align with clinical trial design. ANN NEUROL 20269999:n/a–n/a

Although treatments are available to slow disease progression in some people with typical late‐onset Alzheimer's disease (AD), there are no disease‐modifying treatments for other neurodegenerative pathologies, such as frontotemporal lobar degeneration (FTLD).^1^, ^2^, ^3^ Familial forms of FTLD (f‐FTLD), most often caused by autosomal dominant mutations in the *C9orf72*, *GRN*, or *MAPT* genes, offer unique opportunities for treatment because each mutation involves a single gene and an increasingly well‐delineated pathophysiological cascade.^4^, ^5^ In recent years, several targeted treatments have entered clinical trials for these genetic forms.^2^, ^6^, ^7^ However, age of onset in f‐FTLD varies substantially even among people with the same mutation or within the same family.^8^ This unpredictability may impede selection of appropriate candidates for prevention trials and complicate decisions about when to initiate preventive treatments. For some neurodegenerative disorders, monitoring the status of typically affected brain regions (eg, the hippocampus in AD) has been suggested as a strategy to estimate time to symptom onset.^9^ However, in FTLD, spatial topography of pathology varies even within a genetic group, and monitoring changes in standard regions of interest may miss early signs of emerging neurodegeneration.

Our group previously demonstrated that individualized brain atrophy maps improve prediction of time to onset of dementia in FTLD mutation carriers beyond age alone.^10^ We recently developed a framework designed to improve upon cross‐sectional methodology by applying a Bayesian linear mixed‐effects (BLME)^11^ model to longitudinal MRI scans to track subtle indications of neurodegeneration.^12^ Based on hypothetical^13^ and data‐driven^14^ models of f‐FTLD disease progression showing that acceleration of brain volume loss precedes clinical symptom onset, our method proposes that detection of this acceleration might permit more accurate predictions than established methods. We initially studied amyloid‐positive participants from the Alzheimer's Disease Neuroimaging Initiative (ADNI) cohort who were unimpaired at enrollment and underwent at least 3 longitudinal scans.^12^ Using the first 2 scans from a participant while cognitively intact (henceforth referred to as the “predictor scan pair”), we fit them to a control group's trajectories to estimate individual voxel‐wise rates of volume loss and identified clusters exhibiting accelerated atrophy relative to controls at subsequent timepoints. Both the size of these maps and the speed with which they grew predicted time to dementia conversion in AD more accurately than hippocampal volumes.

In the present study, we extend this framework to f‐FTLD by applying the BLME model to a large cohort of *C9orf72*, *GRN*, or *MAPT* mutation carriers and family controls with longitudinal magnetic resonance imaging (MRI) scans to test the accuracy of predicting conversion to dementia using survival analysis. We compared the performance of this method with other methods, including individualized atrophy maps from cross‐sectional images and regional volumes measured uniformly across participants. To assess its anticipated utility in clinical trial design, we also evaluated its ability in the receiver operating characteristic (ROC) curve analyses to identify individuals who would convert to dementia (Clinical Dementia Rating [CDR] ≥1) within a 24‐month timeframe, a potential proximity marker with which to prioritize presymptomatic participants for clinical trials.

## Methods

### Participants

The present study included 234 carriers of mutations in the *C9orf72*, *GRN*, or *MAPT* genes. Study participants were included from (1) the ARTFL LEFFTDS Longitudinal Frontotemporal Lobar Degeneration (ALLFTD) Research Study,^13^, ^15^ (2) the Genetic Frontotemporal Dementia Initiative (GENFI; data freeze = 7),^16^ and (3) research studies at the UCSF Memory and Aging Center (MAC). ALLFTD and GENFI data have recently been harmonized through the FTD Prevention Initiative (FPI; https://thefpi.org). ALLFTD enrolled participants from 28 sites across the United States and Canada (study enrollment 2015 to present). GENFI enrolled participants from 25 sites across Canada and Europe and spanned 2 study phases: GENFI 1 (2012–2015) and GENFI 2 (2015–2023). Participants were included from both study phases. The University of California – San Francisco (UCSF) MAC participants were enrolled in longitudinal studies of FTLD who had similar brain imaging acquisitions and clinical/genetic data available as in the other 2 studies. All studies enrolled participants with a family history consistent with f‐FTLD; for additional information regarding inclusion/exclusion criteria for each study's recruitment, see Staffaroni et al, 2022.^14^ Protocols at all sites received prior approval by appropriate institutional review boards, and all subjects provided informed consent.

### Clinical and Genetic Assessment

All participants in the ALLFTD, GENFI 2, and MAC studies underwent serial longitudinal examination including neurological history/examination, informant interview, and CDR + National Alzheimer's Coordinating Center (NACC)‐FTLD. The CDR + NACC‐FTLD is a rating scale with 8 domains assessed based on informant report; we used the global score to classify individuals as asymptomatic (0), questionably or mildly symptomatic (0.5), or symptomatic (≥1).^17^, ^18^ Participants enrolled in the GENFI 1 study underwent neurological history/examination, informant interview, and assessment comparable to the CDR + NACC‐FTLD, which assessed whether participants were symptomatic or not in the domains of behavior, neuropsychiatric, language, cognition, and motor functioning. For the present study, individuals were included if they had ≥3 longitudinal MRI scans and were asymptomatic or minimally symptomatic at each of the first 2 scans. Sensitivity analyses were conducted in those who were asymptomatic at both initial timepoints (see 2.3 Neuroimaging below for additional details). A subset of individuals in the study “converted” to dementia over the course of their enrollment, defined as starting with a CDR + NACC‐FTLD of 0 or 0.5 and having a CDR + NACC‐FTLD ≥1 at a subsequent visit (see 2.5 Statistical Analyses for additional details).

ALLFTD and UCSF participants had genetic testing completed at the same laboratory at UCLA using previously published methods.^19^, ^20^ GENFI participants completed genotyping at individual sites.^16^ All participants in this analysis had a pathogenic variation in the *MAPT* or *GRN* genes or a pathogenic expansion in the *C9orf72* gene in themselves or in a family member. Non‐carriers from mutation‐carrying families were used to estimate normative rates of brain volume loss (see details below).

### Neuroimaging Acquisition

ALLFTD/MAC: Participants underwent structural imaging using 3T scanners from 1 of 3 vendors: Siemens, Philips Medical System, or General Electric Medical Systems. A standard T1‐weighted 3D magnetization prepared rapid gradient echo (MPRAGE) sequence was used with the following parameters: 240 × 256 × 256 matrix; approximately 170 slices; voxel size = 1.05 × 1.05 × 1.25 mm^3^; flip angle, TE, and TR varied by vendor. Additional details have been previously published.^21^

GENFI: Participants underwent structural imaging using 3T scanners from Siemens Trio and Skyra, Philips Achieva, and GE Discovery MR750 models. A standard T1‐weighted MPRAGE sequence was used with the following parameters: 256 × 256 × 208 matrix; 208 slices; voxel size = 1.1‐mm isotropic resolution volumetric; flip angle = 8 degrees; TE and TR varied by vendor. Additional details have been previously published.^16^, ^22^

### Image Processing

#### Image Pre‐Processing and Processing

Image processing was completed using previously described methods.^12^ Briefly, all T1‐weighted images were first visually inspected for quality control and images with excessive motion or imaging artifacts were excluded, bias field correction was applied with N3 algorithm, and images were segmented using SPM12 unified segmentation.^23^ This was followed by intra‐subject template creation, within‐subject modulation, customized group template generation within the Large Deformation Diffeomorphic Metric Mapping framework, normalization, and smoothing (8 mm full width half maximum Gaussian kernel), with inspection at every step.^24^, ^25^, ^26^, ^27^ Modulated gray matter maps transformed into the single subject template and warped into the group template were ultimately used in the BLME models. Additionally, we used gray matter maps modulated in the group template to generate atrophy W‐maps and quantify regional volumes (see specific sections below for additional information). Volumetric calculations were performed using FSL and ANTs.^28^, ^29^

#### Bayesian Linear Mixed‐Effects Modeling

Our approach is based on the BLME model described in detail by Ziegler and colleagues.^11^ Our extension of the method to create single‐subject Bayesian predictions of regional gray matter volumes has been previously described in detail.^11^, ^12^ Briefly, we used Bayesian modeling to estimate the degree to which an individual participant's atrophy deviates from their expected trajectory of change. All participants in the study were required to have at least 3 longitudinal MRI timepoints, with at least the first 2 (predictor scan pair) being acquired when they were asymptomatic or questionably/minimally symptomatic (CDR + NACC‐FTLD ≤0.5). Gray matter maps for the predictor scan pair were entered into a BLME model (including that individual and control scans) to quantify the rate of gray matter loss in that participant. This information was then used to predict the individual's expected trajectory of neurodegeneration, assuming continued loss at that rate. Then, the observed volumes in the participant's subsequent MRIs were compared with the predicted volumes in each voxel to identify regions where the observed volume was below the expected volume. Importantly, the predictor scan pair was only used to derive individualized trajectories of change, and all primary analyses across BLME, W‐map, and volumetric methods used subsequent scans after the predictor scan pair.

In these subsequent scans, we used the error function (*erf*), which integrates the Bayesian predictive probability distribution for all possible volumes at each voxel for a given timepoint, to quantify the degree to which each observed value deviated from its expected value. The *erf* ranges from −1 to 1. Voxels with volumes close to the predicted volumes at that timepoint have small *erf* values (near 0), whereas large *erfs* indicate voxels with volumes far from expected values. Because our goal was identifying voxels with volume reductions compared with predictions, we only examined voxels with negative *erf* values. The number of voxels showing unexpectedly low volume was multiplied by voxel size to create a BLME atrophy cluster quantified in cubic millimeters (henceforth referred to as “BLME cluster volume”). The choice of *erf* threshold for creating these maps is arbitrary. Thus, similar to our previous work, we looked for potential effects of threshold by creating maps using *erf* thresholds ranging from −0.7 (which includes volumes closer to their expected value) to −0.999 (only including voxels with volumes very far from expected). All primary findings were seen at nearly all thresholds (see Results). All results are shown in the main manuscript at an *erf* threshold of −0.999, with analyses for additional thresholds displayed in the Supplementary Materials.

#### Atrophy W‐Map Creation

As an alternative approach, we created W‐maps of atrophy, representing gray matter atrophy in standard deviations from the reference group mean. W‐scores were created for each subject at each voxel, adjusting for age, total intracranial volume (TIV), and MRI scanner as covariates. Biological sex was not included as a covariate in w‐score calculation and was instead considered in post hoc sex‐stratified analyses. For additional information, see Staffaroni et al, 2022.^14^ All W‐map results are shown at a whole brain σ threshold of −3.0 (corresponding to a probability of 0.0015 or less of observing this value under a normal distribution) to facilitate comparison with the *erf* threshold of −0.999 (representing voxels where the probability of seeing the observed value is 0.001 or less).

#### Region of Interest Volume Calculation

As a third approach to estimating atrophy, regional volumes were estimated by calculating the mean of gray matter volume in regions of interest (ROIs) from the Desikan‐Killiany cortical atlas,^30^ which was applied to each individual subject's smoothed, modulated, gray matter image. For additional information, see Staffaroni et al, 2022.^14^

### Statistical Analyses

*Survival Analyses*. Metrics from each of the 3 image processing techniques (BLME, atrophy W‐map, and ROI volume) were normalized via z‐score and entered into separate Cox proportional hazard models with time‐dependent predictors to perform survival analysis (using the Python lifelines package [https://zenodo.org/records/3969500]) with imaging metric as predictor, age (and TIV for ROI volume analyses) as covariates, and time to dementia conversion (ie, CDR + NACC‐FTLD ≥ 1) as survival outcome. W‐map and ROI volume values were taken from the same timepoints used for the BLME analysis (ie, not including the 2 baseline scans used to model individual trajectories). Conversion to dementia was the event of interest, whereas non‐converters were censored at their last observation in the study. Resulting hazard ratios (HRs) are interpreted as increased risk of dementia conversion for each one standard deviation increase in the predictor. Due to previously established differences in disease vulnerability based on biological sex,^31^, ^32^ we performed subanalyses for BLME and W‐map models stratified by sex.

#### ROC Curve Analyses

A subset of participants was selected to conduct cross‐sectional analyses assessing the utility of different imaging metrics in classifying individuals who converted versus did not convert to dementia within an approximately 24‐month period to align with the length of a hypothetical clinical trial. To create our dementia converter subset (“24‐month converters”), we first filtered out timepoints on or after the date of conversion (ie, survival date), as well as any scans more than 27 months (24 months + 3 month buffer) prior to the date of conversion. This resulted in our 24‐month converters subset of 11 participants, and, for each of these participants, we only used the imaging timepoint closest to the specified test period (ie, 27 months prior to the date of conversion). To create our non‐converter subset (“24‐month non‐converters”), we used only non‐converters from the main analysis who had an MRI scan at least 24 months prior to the date of last observation in the study. This approach was used to ensure that the non‐converter group consisted of individuals who we were certain did not convert within at least a 24‐month period. This resulted in our 24‐month non‐converters subset of 85 participants, and, for each of these participants, we only used the imaging timepoint closest to the specified test period. Within this new subset of 96 participants, we conducted separate cross‐sectional ROC curve analyses with each imaging metric of interest (BLME cluster volume, atrophy W‐map, and ROI volume) to estimate the area under the curve (AUC) for differentiation of converters from non‐converters. ROC curve analyses were performed separately for each imaging metric of interest in a combined sample of mutation carriers, as well as individually within each genetic mutation. We used Delong's test to compare AUCs of different imaging metrics and assess whether they statistically differed in classifier performance.

## Results

### Demographics

Of the 234 f‐FTLD mutation carriers included in the present study, 96 were *C9orf72* carriers, 76 were *GRN* carriers, and 63 were *MAPT* carriers, with 1 participant having both a *GRN* and *C9orf72* mutation. Of the 234 f‐FTLD mutation carriers, 21 converted to dementia (ie, CDR + NACC‐FTLD ≥ 1), which included 8 *C9orf72* carriers, 4 *GRN* carriers, 8 *MAPT* carriers, and 1 carrier of both a *GRN* and a *C9orf72* mutation. Participant demographics and characteristics for the full sample are presented in Table 1; demographics for the survival (n = 234, 21 converters) and ROC (n = 96, 11 converters) analysis subsets are shown in Supplementary Table S1.

**TABLE 1:** Demographics

	Group			
	All Mutation Carriers	*C9orf72* ^*a*^	*GRN* ^*a*^	*MAPT*
N	234	96	76	63
Baseline age, mean (SD)	47.8 (12.5)	47.5 (12.1)	53.0 (12.4)	41.9 (10.3)
Females, N (%)	133 (56.8)	54 (56.3)	45 (59.2)	35 (55.6)
Dementia converters, N (%)	21 (9.0)	9 (9.4)	5 (6.6)	8 (12.7)
Baseline CDR + NACC‐FTLD				
0	196	75	70	52
0.5	34	18	5	11
Phenotype at conversion^a^				
bvFTD	16	6	3	7
CBS	1	0	0	1
Dementia‐NOS	1	1	0	0
FTD‐ALS	1	1	0	0
PPA	2	1	2	0
Imaging metric,^b^ mean (SD), [min, max]				
BLME cluster volume, mm^3^	1,675.74 (6,264.74), [0, 53,997]	1,488.49 (6,983.73), [0, 53,997]	1,291.95 (6,342.89), [0, 53,997]	3,254.54 (8,091.86), [0, 43,431]
W‐map cluster volume, mm^3^	56,189.68 (68,114.01), [647, 431,625]	66,841.23 (76,199.76), [647, 431,625]	39,047.97 (54,803.12), [1768, 269,586]	63,992.32 (71,017.41), [2,604, 320,552]
Frontal ROI volume, mm^3^	91,162.52 (12,353.04), [57,725.63, 136,420.54]	89,824.60 (12,170.10), [63,961.28, 127,875.04]	91,815.53 (12,481.81), [60,471.29, 122,034.94]	91,982.79 (12,882.58), [57,725.63, 136,420.54]
Temporal ROI volume, mm^3^	63,783.90 (7,947.62), [41,542.30, 87,446.59]	63,288.63 (8,052.18), [47,873.03, 87,446.59]	63,799.10 (7,910.31), [45,232.59, 84,517.43]	64,394.90 (7,910.89), [41,542.30, 84,691.58]

^a^ One converter was a carrier of both a *GRN* and a *C9orf72* mutation.

^b^ Imaging metrics are provided for the most recent timepoint and at thresholds of *erf* = 0.999 for BLME and *σ* = 3.0 for W‐map.

ALS = amyotrophic lateral sclerosis; BLME = Bayesian linear mixed‐effects; bvFTD = behavioral variant frontotemporal dementia; CBS = corticobasal syndrome; CDR = Clinical Dementia Rating scale; FTD = frontotemporal dementia; FTLD = frontotemporal lobar degeneration; NACC = National Alzheimer's Coordinating Center; NOS = not otherwise specified; PPA = primary progressive aphasia; ROI = region of interest; SD = standard deviation.

### Survival Analyses

**FIGURE 1:**
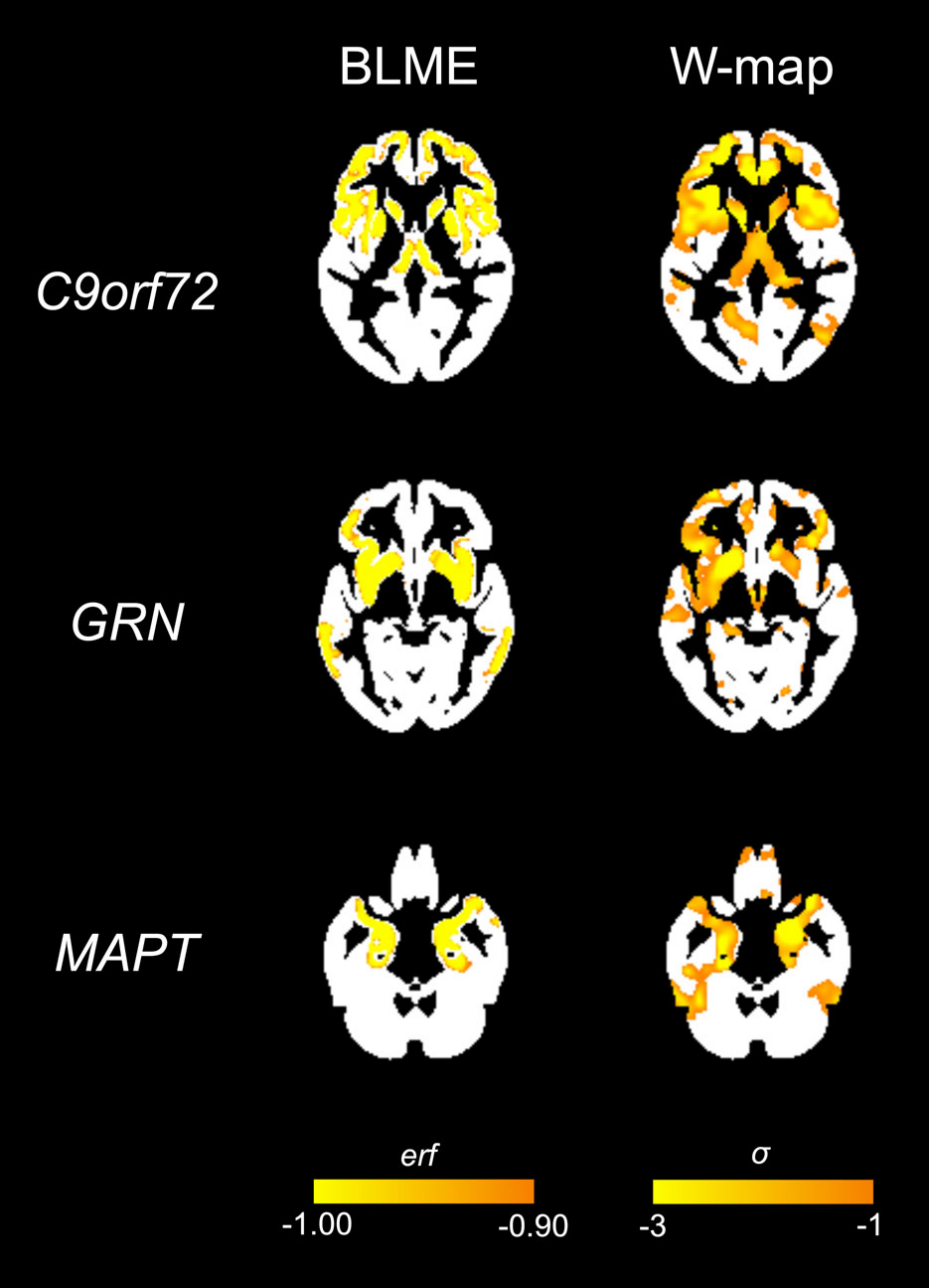
Example BLME cluster volumes and W‐maps from a specific individual for each gene overlaid on template brains. BLME = Bayesian linear mixed‐effect. [Color figure can be viewed at www.annalsofneurology.org]

Examples of single‐subject images of BLME cluster volumes and W‐map cluster volumes are shown for each mutation in Figure 1. At an *erf* threshold of 0.999, BLME cluster volume significantly predicted conversion to dementia in f‐FTLD mutation carriers overall (n = 234, HR = 2.46, 95% confidence interval [CI] = 1.89–3.20, *p* = 2.5 × 10^−11;^ Table 2). At a comparable threshold of *σ* = 3.0, overall W‐map cluster volume also significantly predicted conversion to dementia across carrier groups (n = 234, HR = 3.00, 95% CI = 1.80–3.65, *p* = 2.0 × 10^−6^). At the same 0.999 threshold, BLME cluster volume also significantly predicted conversion to dementia separately in each f‐FTLD mutation, with the largest effect size observed in *MAPT* (n = 63, HR = 2.89, 95% CI = 1.46–5.72, *p* = 0.0022), followed by *GRN* (n = 76, HR = 2.67, 95% CI = 1.55–4.59, *p* = 0.0004) and *C9orf72* (n = 96, HR = 1.77, 95% CI = 1.37–2.28, *p* = 0.00001; see Table 2). Comparable HRs were observed when examining W‐map cluster volume or ROI volumes (frontal and temporal lobes) as predictors (see Table 2). Using alternative BLME *erf* thresholds of 0.99, 0.9, 0.8, and 0.7, as well as alternative W‐map σ thresholds of 2.5, 2.0, 1.5, and 1.0, both BLME cluster volume and W‐map cluster volume still significantly predicted conversion to dementia across all f‐FTLD mutation carriers and within each individual mutation (Supplementary Materials). Sensitivity analyses limited to only those who were asymptomatic at baseline (ie, CDR + NACC FTLD = 0) showed significant prediction of conversion to dementia across all carriers for both BLME cluster volume (n = 165, HR = 3.23, 95% CI = 1.79–5.85, *p* = 0.00011) and W‐map cluster volume (n = 165, HR = 9.51, 95% CI = 2.68–33.72, *p* = 0.00049). In sex‐stratified models, BLME cluster volume significantly predicted conversion to dementia in a combined group of f‐FTLD mutation carriers in female subjects only (n = 133, HR = 2.36, 95% CI = 1.76–3.17, *p* = 1.1 × 10^−8^) and in male subjects only (n = 101, HR = 2.69, 95% CI = 1.05–6.88, *p* = 0.0385). When examining W‐map cluster volume as predictor, prediction of dementia conversion was significant in female subjects (n = 133, HR = 3.34, 95% CI = 2.11–5.27, *p* = 2.3 × 10^−7^) but not in male subjects (n = 101, HR = 1.93, 95% CI = 0.60–6.26, *p* = 0.27), although the effect was in the expected direction for male subjects.

**TABLE 2:** Survival Analysis Results

	BLME^a^	W‐map^b^	Frontal ROI	Temporal ROI
All genes (n = 234)	2.46 [1.89, 3.20] *p* = 2.5 × 10^−11^	3.00 [1.91, 4.71] *p* = 2.0 × 10^−6^	2.63 [1.75, 3.98] *p* = 3.9 × 10^−6^	2.74 [1.64, 4.57] *p* = 0.00012
*C9orf72* (n = 96)	1.77 [1.37, 2.28] *p* = 0.00001	1.62 [1.10, 2.39] *p* = 0.014	2.97 [1.63, 5.41] *p* = 0.00036	1.72 [0.80, 3.68] *p* = 0.16
*GRN* (n = 76)	2.67 [1.55, 4.59] *p* = 0.0004	14.35 [1.47, 139.87] *p* = 0.022	3.46 [1.15, 10.34] *p* = 0.027	4.90 [0.89, 26.95] *p* = 0.07
*MAPT* (n = 63)	2.89 [1.46, 5.72] *p* = 0.0022	3.06 [1.66, 5.62] *p* = 0.0003	2.03 [1.06, 3.91] *p* = 0.033	2.91 [1.50, 5.64] *p* = 0.0016

Hazard ratio (HR) and 95% confidence intervals (in brackets) are displayed for BLME cluster volume, W‐map cluster volume, and ROI volumes as predictors of conversion to dementia in survival analyses.

^a^ BLME values are displayed at a threshold of *erf* = 0.999.

^b^ W‐map values are displayed at a threshold of σ = 3.0.

BLME = Bayesian linear mixed‐effects; ROI = regions of interest.

### ROC Curve Analyses

**FIGURE 2:**
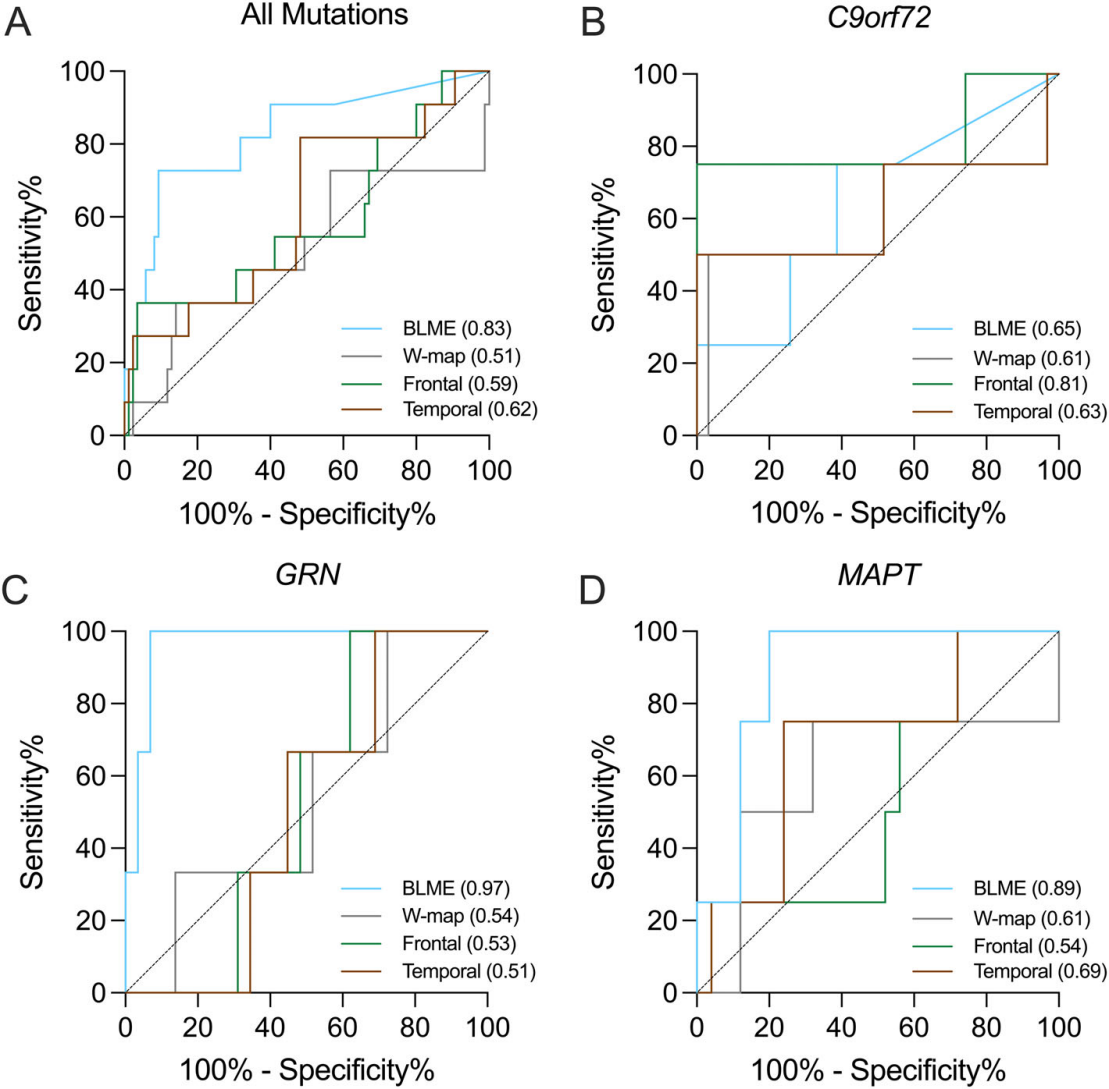
ROC curves for predicting conversion to dementia within 24 months. ROC curves and AUCs displayed for BLME (at *erf* threshold of 0.999), W‐map (at *σ* = 3.0), frontal lobe ROI volume, and temporal lobe ROI volume across (A) all mutation carriers, (B) *C9orf72* carriers, (C) *GRN* carriers, and (D) *MAPT* carriers. AUC = area under the curve; BLME = Bayesian linear mixed‐effect; ROC = receiver‐operating characteristic. [Color figure can be viewed at www.annalsofneurology.org]

BLME cluster volume demonstrated robust accuracy in differentiating converters from non‐converters at an *erf* threshold of 0.999 (AUC = 0.83, 95% CI = 0.68–0.97, *p* = 0.0004), as well as at other *erf* thresholds (Table 3). BLME cluster volume performed better in discriminating between converters versus non‐converters compared to W‐map cluster volume at *σ* = 3.0 (n = 96, AUC = 0.51, 95% CI = 0.30–0.73, *p* = 0.89, Delong's test Z = 2.68, *p* = 0.007), frontal lobe volume (n = 96, AUC = 0.59, 95% CI = 0.39–0.79, *p* = 0.34, Delong's test Z = 1.59, *p* = 0.11), or temporal lobe volume (n = 96; AUC = 0.62, 95% CI = 0.43–0.80, *p* = 0.21, Delong's test Z = 1.55, *p* = 0.12; see Table 3 and Fig 2). The highest predictive accuracy for BLME cluster volume was observed in *GRN* (n = 32, AUC = 0.97, 95% CI = 0.90–1.00, *p* = 0.0088) followed by *MAPT* (n = 29, AUC = 0.89, 95% CI = 0.77–1.00, *p* = 0.014), whereas the AUC for *C9orf72* was not statistically significant (n = 35, AUC = 0.65, 95% CI = 0.34–0.95, *p* = 0.35; see Table 3 and Fig 2). Supporting the robustness of this approach, similar results were observed across thresholds (Supplementary Materials).

**TABLE 3:** Accuracy in Predicting Conversion to Dementia Within 24 Months

	BLME *erf* = 0.999	W‐map *σ* = 3.0	Frontal ROI	Temporal ROI
All genes (n = 96)	0.83 [0.68, 0.97] *p* = 0.0004	0.51 [0.30, 0.73] *p* = 0.89	0.59 [0.39, 0.79] *p* = 0.34	0.62 [0.43, 0.80] *p* = 0.21
*C9orf72* (n = 35)	0.65 [0.34, 0.95] *p* = 0.35	0.61 [0.22, 1.00] *p* = 0.47	0.81 [0.50, 1.00] *p* = 0.043	0.63 [0.23, 1.00] *p* = 0.41
*GRN* (n = 32)	0.97 [0.90, 1.00] *p* = 0.0088	0.54 [0.23, 0.85] *p* = 0.82	0.53 [0.31, 0.75] *p* = 0.87	0.51 [0.28, 0.73] *p* = 0.97
*MAPT* (n = 29)	0.89 [0.77, 1.00] *p* = 0.014	0.61 [0.24, 0.98] *p* = 0.49	0.54 [0.25, 0.83] *p* = 0.80	0.69 [0.41, 0.97] *p* = 0.23

Area under the curve (AUC) and 95% confidence intervals (in brackets) are displayed for BLME cluster volume, W‐map cluster volume, and ROI volumes showing utility of discrimination between participants who converted versus did not convert to dementia within 24 months. BLME = Bayesian linear mixed‐effects; ROI = regions of interest.

## Discussion

This study demonstrated the utility of Bayesian‐modeled individualized atrophy scores in predicting progression to dementia among f‐FTLD mutation carriers. We expand on prior work from our group that has (1) used cross‐sectional atrophy‐based scores to predict dementia in f‐FTLD,^10^ and (2) used a similar Bayesian‐modeled atrophy score approach to predict conversion to AD dementia.^10^, ^12^ We extend those findings by using longitudinal neuroimaging to quantify the extent to which an individual's atrophy diverges from their expected trajectory of volume loss and show that these estimates predict conversion to dementia in presymptomatic f‐FTLD mutation carriers. ROC curve analyses suggest BLME cluster volume can identify participants at higher risk of developing dementia within a 24‐month period, highlighting the potential to inform clinical trial design. In 24‐month analyses, BLME cluster volumes that leveraged longitudinal data outperformed cross‐sectional estimates (using both W‐maps and standard ROI approaches) for distinguishing between converters and non‐converters, supporting the hypothesis that estimates of atrophy that consider information from “run‐in” scans may better capture symptom onset than traditional atrophy maps. Our BLME approach offers unique advantages over classic linear models for predicting future outcomes. Whereas frequentist methods provide point estimates and associated uncertainty for current observations, the Bayesian approach yields a prediction framework for all possible future observations, allowing probabilistic statements about them. In addition, the Bayesian framework includes prior distributions and naturally incorporates regularization to reduce overfitting. Together, our results position BLME cluster volume as a potentially useful biomarker for early‐stage dementia detection in f‐FTLD that may support personalized intervention strategies and improve clinical trial design.

Our findings contribute to the growing literature on the utility of structural MRI in predicting disease onset and symptom progression in f‐FTLD and related neurodegenerative disorders.^10^, ^14^, ^21^, ^33^, ^34^, ^35^, ^36^, ^37^ Prior work from our group found that regional brain atrophy using standard W‐map quantification, along with plasma NfL, emerged as the first observable abnormal biomarkers in f‐FTLD.^14^ Recent work has suggested initial hemispheric lateralization of brain atrophy is important in f‐FTLD progression, which may be captured by using person‐specific atrophy trajectories.^38^ Our study builds on these prior findings by using a data‐driven, probabilistic BLME framework to quantify individual deviations from expected trajectories of brain atrophy. Our subgroup analyses, although limited by smaller sample sizes, suggested the greatest utility of BLME cluster volume was for prediction in *MAPT* and *GRN* mutations rather than *C9orf72*. One possible explanation is the relatively more severe and rapid acceleration of neurodegeneration associated with these mutations.^33^, ^34^ In contrast, *C9orf72* mutation carriers tend to be more heterogeneous in their presentations (eg, behavioral variant frontotemporal dementia [bvFTD] and amyotrophic lateral sclerosis [ALS]) and have more prolonged and relatively more linear trajectories of volume loss.^14^, ^35^, ^39^, ^40^ Linear trajectories may be particularly problematic for this methodology given the reliance on detecting deviations from one's own trajectory, which would thus be insensitive to linear volume loss. This explanation remains speculative, and future efforts will systematically examine how linearity may affect the utility of BLME methods. Despite these mutation‐specific differences, BLME‐derived atrophy volumes consistently predicted conversion across all genetic variants, providing robust evidence for structural MRI as a prodromal biomarker that may transcend the clinical heterogeneity of f‐FTLD. Prior work has highlighted potential differences in vulnerability to neurodegenerative diseases between women and men,^31^, ^32^ prompting our exploratory sex‐stratified analyses. BLME cluster volume significantly predicted dementia conversion in both sexes, with similar effect sizes; *p* values were smaller in women, potentially reflecting the larger sample size of female subjects. In contrast, W‐map cluster volume showed larger effect sizes in women and was significant only in female subjects, potentially suggesting that the BLME approach is more robust to sex differences. Alternatively, TIV is a covariate in the w‐score adjustments, and given the strong association of TIV with sex, this adjustment may remove important disease related effects and reduce predictive accuracy. Definitive conclusions about sex‐specific effects cannot yet be drawn given the small sample sizes here, and future studies with adequately powered cohorts will be critical to determine whether sex differences exist across methodologies and mutation carrier groups.

The predictive utility of BLME‐derived cluster volume has potential implications for clinical trial design, as identifying mutation carriers with a high probability of developing dementia within 24 months allows for more targeted participant selection and may in turn improve trial efficiency. This is crucial in f‐FTLD trials, which are difficult to power due to rarity and clinical heterogeneity.^14^, ^41^ A future direction is to not only predict when someone will become clinically symptomatic, but the type of clinical symptoms they may first experience based on prodromal brain atrophy patterns. Inclusion of quantitative clinical and cognitive outcomes would complement structural imaging and may provide greater sensitivity to detect subtle clinical changes in the asymptomatic to minimally symptomatic transition stage. Combining multimodal data (eg, cognitive and imaging) may be particularly helpful in *C9orf72* carriers given the clinical heterogeneity and relatively prolonged trajectories often observed in this group. Future studies may also include additional genetic modifiers known to influence disease progression, such as *TMEM106B*, to refine risk stratification and increase the applicability of our method to broader populations. Future studies that incorporate cortical thickness metrics, in both combined models and sex‐stratified models, may provide complementary insights into neurodegenerative patterns in f‐FTLD, as cortical thickness measures can capture subtle changes in the cerebral cortex that may not be fully reflected in volumetric measures.

BLME cluster volume may be a promising marker for predicting disease onset, but there are several remaining challenges. Although AUCs for BLME cluster volumes were more robust than other metrics, HRs from longitudinal survival analyses were largely comparable across BLME cluster volume, atrophy W‐map, and ROI volume as predictors. Thus, the BLME method may not outperform other structural imaging methods, highlighting the importance of avoiding overinterpretation of differences. Future studies incorporating direct comparisons with other established longitudinal MRI methods (eg, tensor‐based morphometry and boundary‐shift integral) will be important in determining the unique value of BLME metrics. Similarly, there are inherent limitations in comparing the cross‐sectional 24‐month ROC curve analyses to the longitudinal survival analyses due to the different samples used (n = 96 vs n = 234, respectively) and the different data used in each model (single timepoint vs multiple timepoints, respectively). Additionally, the ROC curve analyses involved a subsample of participants, introducing potential bias and limiting generalizability. Our BLME method is also more resource intensive, as a minimum of 3 longitudinal MRI scans are required. Requiring a minimum of 3 MRI scans may increase the financial and time burden for researchers and participants, and it is important to consider whether the advantages of a BLME approach over simpler approaches outweigh the costs. In particular, percentile maps are generated in workflows of numerous existing clinical research and trial settings, and we envision a detailed evaluation of cost‐effectiveness, feasibility, and clinical integration of this approach versus our BLME metric as an important future direction. Threshold selection can influence predictive performance and remains an area for methodological refinement. In this study, we explored a range of thresholds based on prior applications in AD,^12^ with results demonstrating consistent significance across this range. Future work will explore more granular, data‐driven optimization approaches (eg, ROC‐based or cross‐validated selection) to further enhance robustness and reproducibility. Furthermore, our study only examined negative *erf* values, as the goal of our study was to focus on regions that were far from the expected value, and future efforts may also examine the predictive value of positive *erf* values (ie, areas that are a less‐than‐expected decrease). The present study, which sampled across North American and European f‐FTLD research networks, had a relatively large longitudinal sample given the rarity of the disease and the restricted inclusion criteria, but sample sizes were nonetheless relatively small for each genetic group. We included some individuals with mild symptoms (CDR + NACC‐FTLD = 0.5) due to sample size considerations, but also because mild symptoms are not always indicators of neurodegeneration and participants with mild symptoms can revert to being asymptomatic and may remain so for a number of years.^42^ Therefore, disease‐specific, biomarker‐based metrics of neurodegeneration are very important to assess in these cases. The expected effect of including these cases would be to decrease the BLME's sensitivity to detecting atrophy at future timepoints, as atrophy rates for some of these participants may have already begun accelerating. As recruitment of asymptomatic members of families affected by these mutations become more common, regular scans well before disease pathophysiology affects brain volume may become more available.

Our study demonstrates that BLME cluster volume predicts dementia conversion in f‐FTLD mutation carriers, with particular utility in the *GRN* and *MAPT* mutations. This innovative methodology, which can be combined with other biomarkers (eg, plasma neurofilament light chain [NfL] and positron emission tomography [PET] imaging), may help with refining participant selection and trial outcome optimization to tailor mutation‐specific therapeutics (Supplementary Fig S2).

## Author Contributions

S.D., D.L., Y.C., H.J.R., and A.M.S. contributed to the conception and design of the study; S.D., D.L., Y.C., H.J.R., and A.M.S. contributed to the acquisition and analysis of data; S.D., D.L., Y.C., H.J.R., and A.M.S. contributed to drafting the text and preparing the figures.

## Potential Conflicts of Interest

Nothing to report.

## Supporting information


**Supplementary Data S1.** Supplementary Information.

## Data Availability

Qualified researchers may request access to deidentified data for this project through the FTD Prevention Initiative (https://thefpi.org/).

## References

[1] Boxer AL , Sperling R . Accelerating Alzheimer's therapeutic development: the past and future of clinical trials. Cell 2023;186:4757–4772. 37848035 10.1016/j.cell.2023.09.023 PMC10625460

[2] Tsai RM , Boxer AL . Therapy and clinical trials in frontotemporal dementia: past, present, and future. J Neurochem 2016;138:211–221. 27306957 10.1111/jnc.13640 PMC5217534

[3] Knopman DS , Roberts RO . Estimating the number of persons with frontotemporal lobar degeneration in the US population. J Mol Neurosci 2011;45:330–335. 21584654 10.1007/s12031-011-9538-y PMC3208074

[4] Greaves CV , Rohrer JD . An update on genetic frontotemporal dementia. J Neurol 2019;266:2075–2086. 31119452 10.1007/s00415-019-09363-4 PMC6647117

[5] Roberson ED , Hesse JH , Rose KD , et al. Frontotemporal dementia progresses to death faster than Alzheimer disease. Neurology 2005;65:719–725. 16157905 10.1212/01.wnl.0000173837.82820.9f

[6] Boeve BF , Boxer AL , Kumfor F , et al. Advances and controversies in frontotemporal dementia: diagnosis, biomarkers, and therapeutic considerations. Lancet Neurol 2022;21:258–272. 35182511 10.1016/S1474-4422(21)00341-0

[7] Neylan KD , Miller BL . New approaches to the treatment of frontotemporal dementia. Neurotherapeutics 2023;20:1055–1065. 37157041 10.1007/s13311-023-01380-6 PMC10457270

[8] Barbier M , Camuzat A , Houot M , et al. Factors influencing the age at onset in familial frontotemporal lobar dementia. Neurol Genet 2017;3:e203. 29264395 10.1212/NXG.0000000000000203 PMC5730818

[9] Csernansky JG , Wang L , Swank J , et al. Preclinical detection of Alzheimer's disease: hippocampal shape and volume predict dementia onset in the elderly. Neuroimage 2005;25:783–792. 15808979 10.1016/j.neuroimage.2004.12.036

[10] Staffaroni AM , Cobigo Y , Goh SYM , et al. Individualized atrophy scores predict dementia onset in familial frontotemporal lobar degeneration. Alzheimers Dement 2020;16:37–48. 31272932 10.1016/j.jalz.2019.04.007 PMC6938544

[11] Ziegler G , Penny WD , Ridgway GR , et al. Estimating anatomical trajectories with Bayesian mixed‐effects modeling. Neuroimage 2015;121:51–68. 26190405 10.1016/j.neuroimage.2015.06.094 PMC4607727

[12] Cobigo Y , Goh MS , Wolf A , et al. Detection of emerging neurodegeneration using Bayesian linear mixed‐effect modeling. Neuroimage Clin 2022;36:103144. 36030718 10.1016/j.nicl.2022.103144 PMC9428846

[13] Rosen HJ , Boeve BF , Boxer AL . Tracking disease progression in familial and sporadic frontotemporal lobar degeneration: recent findings from ARTFL and LEFFTDS. Alzheimers Dement 2020;16:71–78. 31914219 10.1002/alz.12004 PMC6953606

[14] Staffaroni AM , Quintana M , Wendelberger B , et al. Temporal order of clinical and biomarker changes in familial frontotemporal dementia. Nat Med 2022;28:2194–2206. 36138153 10.1038/s41591-022-01942-9 PMC9951811

[15] Boeve B , Bove J , Brannelly P , et al. The longitudinal evaluation of familial frontotemporal dementia subjects protocol: framework and methodology. Alzheimers Dement 2020;16:22–36. 31636026 10.1016/j.jalz.2019.06.4947 PMC6949411

[16] Rohrer JD , Nicholas JM , Cash DM , et al. Presymptomatic cognitive and neuroanatomical changes in genetic frontotemporal dementia in the genetic frontotemporal dementia initiative (GENFI) study: a cross‐sectional analysis. Lancet Neurol 2015;14:253–262. 25662776 10.1016/S1474-4422(14)70324-2 PMC6742501

[17] Miyagawa T , Brushaber D , Syrjanen J , et al. Use of the CDR® plus NACC FTLD in mild FTLD: data from the ARTFL/LEFFTDS consortium. Alzheimers Dement 2020;16:79–90. 31477517 10.1016/j.jalz.2019.05.013 PMC6949373

[18] Miyagawa T , Brushaber D , Syrjanen J , et al. Utility of the global CDR® plus NACC FTLD rating and development of scoring rules: data from the ARTFL/LEFFTDS consortium. Alzheimers Dement 2020;16:106–117. 31914218 10.1002/alz.12033 PMC7202045

[19] Gefen T , Ahmadian SS , Mao Q , et al. Combined pathologies in FTLD‐TDP types a and C. J Neuropathol Exp Neurol 2018;77:405–412. 29584904 10.1093/jnen/nly018 PMC6019001

[20] Ramos EM , Dokuru DR , Van Berlo V , et al. Genetic screening of a large series of north American sporadic and familial frontotemporal dementia cases. Alzheimers Dement 2020;16:118–130. 31914217 10.1002/alz.12011 PMC7199807

[21] Olney NT , Ong E , Goh SYM , et al. Clinical and volumetric changes with increasing functional impairment in familial frontotemporal lobar degeneration. Alzheimers Dement 2020;16:49–59. 31784375 10.1016/j.jalz.2019.08.196 PMC6988137

[22] Russell LL , Greaves CV , Bocchetta M , et al. Social cognition impairment in genetic frontotemporal dementia within the GENFI cohort. Cortex 2020;133:384–398. 33221702 10.1016/j.cortex.2020.08.023 PMC7754789

[23] Ashburner J , Friston KJ . Unified segmentation. Neuroimage 2005;26:839–851. 15955494 10.1016/j.neuroimage.2005.02.018

[24] Sled JG , Zijdenbos AP , Evans AC . A nonparametric method for automatic correction of intensity nonuniformity in MRI data. IEEE Trans Med Imaging 1998;17:87–97. 9617910 10.1109/42.668698

[25] Ashburner J . A fast diffeomorphic image registration algorithm. Neuroimage 2007;38:95–113. 17761438 10.1016/j.neuroimage.2007.07.007

[26] Ashburner J , Ridgway GR . Symmetric diffeomorphic modeling of longitudinal structural MRI. Front Neurosci 2012;6:197. 23386806 10.3389/fnins.2012.00197 PMC3564017

[27] Fonov V , Evans A , McKinstry R , et al. Unbiased nonlinear average age‐appropriate brain templates from birth to adulthood. Neuroimage 2009;47:S102.

[28] Avants BB , Tustison NJ , Stauffer M , et al. The insight ToolKit image registration framework. Front Neuroinform 2014;8:44. 24817849 10.3389/fninf.2014.00044 PMC4009425

[29] Smith SM , Jenkinson M , Woolrich MW , et al. Advances in functional and structural MR image analysis and implementation as FSL. Neuroimage 2004;23:S208–S219. 15501092 10.1016/j.neuroimage.2004.07.051

[30] Desikan RS , Ségonne F , Fischl B , et al. An automated labeling system for subdividing the human cerebral cortex on MRI scans into gyral based regions of interest. Neuroimage 2006;31:968–980. 16530430 10.1016/j.neuroimage.2006.01.021

[31] Illán‐Gala I , Casaletto KB , Borrego‐Écija S , et al. Sex differences in the behavioral variant of frontotemporal dementia: a new window to executive and behavioral reserve. Alzheimers Dement 2021;17:1329–1341. 33590953 10.1002/alz.12299 PMC8364861

[32] Garcia Castro J , Rubio‐Guerra S , Casaletto KB , et al. Sex differences in the executive and behavioral reserve of autosomal dominant frontotemporal dementia. Alzheimers Dement 2025;21:e70070. 40277045 10.1002/alz.70070 PMC12022895

[33] Whitwell JL , Boeve BF , Weigand SD , et al. Brain atrophy over time in genetic and sporadic frontotemporal dementia: a study of 198 serial magnetic resonance images. Eur J Neurol 2015;22:745–752. 25683866 10.1111/ene.12675 PMC4390434

[34] Rohrer JD , Ridgway GR , Modat M , et al. Distinct profiles of brain atrophy in frontotemporal lobar degeneration caused by progranulin and tau mutations. Neuroimage 2010;53:1070–1076. 20045477 10.1016/j.neuroimage.2009.12.088 PMC2941039

[35] Staffaroni AM , Goh SYM , Cobigo Y , et al. Rates of brain atrophy across disease stages in familial frontotemporal dementia associated with MAPT, GRN, and C9orf72 pathogenic variants. JAMA Netw Open 2020;3:1–17. 10.1001/jamanetworkopen.2020.22847 PMC7593814 33112398

[36] Pankov A , Binney RJ , Staffaroni AM , et al. Data‐driven regions of interest for longitudinal change in frontotemporal lobar degeneration. Neuroimage Clin 2016;12:332–340. 27547726 10.1016/j.nicl.2015.08.002 PMC4983147

[37] Street D , Bevan‐Jones WR , Malpetti M , et al. Structural correlates of survival in progressive supranuclear palsy. Parkinsonism Relat Disord 2023;116:105866. 37804622 10.1016/j.parkreldis.2023.105866 PMC7615224

[38] Borrego‐Ecija S , Juncà‐Parella J , Vandebergh M , et al. Association of Initial Side of brain atrophy with clinical features and disease progression in patients with GRN frontotemporal dementia. Neurology 2024;103:e209944. 39527772 10.1212/WNL.0000000000209944 PMC11558542

[39] Yokoyama JS , Sirkis DW , Miller BL . C9ORF72 hexanucleotide repeats in behavioral and motor neuron disease: clinical heterogeneity and pathological diversity. Am J Neurodegener Dis 2014;3:1–18. 24753999 PMC3986607

[40] Lee SE , Sias AC , Mandelli ML , et al. Network degeneration and dysfunction in presymptomatic C9ORF72 expansion carriers. Neuroimage Clin 2017;14:286–297. 28337409 10.1016/j.nicl.2016.12.006 PMC5349617

[41] Feldman HH , Cummings JL , Boxer AL , et al. A framework for translating tauopathy therapeutics: drug discovery to clinical trials. Alzheimers Dement 2024;20:8129–8152. 39316411 10.1002/alz.14250 PMC11567863

[42] Bondi MW , Edmonds EC , Jak AJ , et al. Neuropsychological criteria for mild cognitive impairment improves diagnostic precision, biomarker associations, and progression rates. J Alzheimer's Dis 2014;42:275–289. 24844687 10.3233/JAD-140276 PMC4133291

