# Supplementary material for: Individualized Atrophy‐Based Prediction of Dementia Progression in Familial Frontotemporal Lobar Degeneration With Bayesian Linear Mixed‐Effects Modeling
Source: Ann Neurol. 2026 Jan 29;99(5):1315–26. doi: 10.1002/ana.78167 (PMC12950279; doi:10.1002/ana.78167)
Supplement: Supplementary file 1 — Supplementary Data S1. Supplementary Information. [file ANA-99-1315-s001.docx]

Supplementary Materials

Supp Methods

Supp Table 1.

OVERALL SURVIVAL ANALYSIS

|  | Group |  |  |  |
| --- | --- | --- | --- | --- |
|  | All Mutation Carriers | *C9orf72* | *GRN* | *MAPT* |
| Demographics |  |  |  |  |
| *n* | 234 | 96 | 76 | 63 |
| Baseline age*, mean (SD) | 47.8 (12.5) | 47.5 (12.1) | 53.0 (12.4) | 41.9 (10.3) |
| Females, N (%) | 133 (56.8%) | 54 (56.3%) | 45 (59.2%) | 35 (55.6%) |
| Dementia converters, N (%) | 21 (9.0%) | 9 (9.4%) | 5 (6.6%) | 8 (12.7%) |

24mo CONVERSION ANALYSIS

|  | Group |  |  |  |
| --- | --- | --- | --- | --- |
|  | All Mutation Carriers | *C9orf72* | *GRN* | *MAPT* |
| Demographics |  |  |  |  |
| *n* | 96 | 35 | 32 | 29 |
| Baseline age*, mean (SD) | 47.6 (12.3) | 46.2 (11.0) | 54.2 (12.9) | 42.1 (9.9) |
| Females, N (%) | 56 (58.3%) | 23 (65.7%) | 18 (56.3%) | 15 (51.7%) |
| Dementia converters, N (%) | 11 (11.5%) | 4 (11.4%) | 3 (9.4%) | 4 (13.8%) |

*Age at baseline of first timepoint entered into survival analysis

Supp Table 2. All values for Survival Analyses

| *erf* |  |  |  |  |  |
| --- | --- | --- | --- | --- | --- |
| Threshold | 0.7 | 0.8 | 0.9 | 0.99 | 0.999 |
| All Genes  n = 234 | 2.46 [1.78, 3.41]  *P*=6.0x10^-8^ | 2.43 [1.80, 3.26] P=2.0x10^-9^ | 2.32 [1.78, 3.02] P=4.0x10^-10^ | 2.19 [1.73, 2.79] P=1.0x10^-10^ | 2.46 [1.89, 3.20] P=3.0x10^-11^ |
| C9*orf72*  n = 96 | 1.99 [1.30, 3.03]  *P*=0.0014 | 1.97 [1.36, 2.87] P=0.0004 | 1.94 [1.39, 2.72] P=0.0001 | 1.85 [1.39, 2.47] P=0.00002 | 1.77 [1.37, 2.28] P=0.00001 |
| *GRN*  n = 76 | 3.75 [1.69, 8.35]  *P*=0.0012 | 3.40 [1.67, 6.94] P=0.0008 | 3.13 [1.64, 5.97] P=0.0006 | 2.81 [1.58, 4.98] P=0.0004 | 2.67 [1.55, 4.59] P=0.0004 |
| *MAPT*  n = 63 | 2.76 [1.41, 5.38]  *P*=0.0029 | 2.54 [1.40, 4.61] P=0.0021 | 2.30 [1.37, 3.88] P=0.0017 | 2.15 [1.31, 3.52] P=0.0025 | 2.89 [1.46, 5.72] P=0.0022 |

| *W-map* |  |  |  |  |  |
| --- | --- | --- | --- | --- | --- |
| Threshold | 1.0 | 1.5 | 2.0 | 2.5 | 3.0 |
| All Genes  n = 234 | 2.35 [1.58, 3.50]  P=4.9x10^-6^ | 2.36 [1.59, 3.51]  P=3.1x10^-6^ | 2.49 [1.66, 3.71]  P=1.1x10^-6^ | 2.66 [1.75, 4.04]  P=4.4x10^-7^ | 3.00 [1.91, 4.71] P=2.0x10^-6^ |
| C9*orf72*  n = 96 | 1.75 [1.07, 2.86]  P=0.025 | 1.73 [1.07, 2.79]  P=0.024 | 1.71 [1.09, 2.70]  P=0.020 | 1.69 [1.10,2.58]  P=0.017 | 1.62 [1.10, 2.39] P=0.014 |
| *GRN*  n = 76 | 4.21 [1.06, 16.68]  P=0.041 | 4.40 [1.10, 17.55]  P=0.036 | 5.23 [1.17, 23.42]  P=0.031 | 7.03 [1.25, 39.55]  P=0.027 | 14.35 [1.47,139.87]  P=0.022 |
| *MAPT*  n = 63 | 3.26 [1.65, 6.44]  P=0.0007 | 3.30 [1.68, 6.48]  P=0.0005 | 3.28 [1.70, 6.33]  P=0.0004 | 3.21 [1.70, 6.06]  P=0.0003 | 3.06 [1.66, 5.62] P=0.0003 |

|  |  |  |  |  |  |
| --- | --- | --- | --- | --- | --- |
| *ROI volume* | Frontal | Temporal | Medial Temporal | Insula | Thalamus |
| All Genes | 2.63 [1.75, 3.98]  P=3.9x10^-6^ | 2.74 [1.64, 4.57]  P=0.00012 | 2.18 [1.57, 3.03]  P=3.9x10^-6^ | 2.81 [1.81, 4.38]  P=4.7x10^-6^ | 1.54 [1.09, 2.18]  P=0.014 |
| C9*orf72*  n = 96 | 2.97 [1.63, 5.41]  P=0.00036 | 1.72 [0.80, 3.68]  P=0.16 | 1.34 [0.68, 2.62]  P=0.39 | 1.90 [0.97, 3.69]  P=0.06 | 1.29 [0.65, 2.57]  P=0.46 |
| *GRN*  n = 76 | 3.46 [1.15, 10.34]  P=0.027 | 4.90 [0.89, 26.95]  P=0.067 | 3.40 [1.18, 9.84]  P=0.024 | 1.77 [0.77, 4.06]  P=0.18 | 3.87 [0.98, 15.24]  P=0.053 |
| *MAPT*  n = 63 | 2.03 [1.06, 3.91]  P=0.033 | 2.91 [1.50, 5.64]  P=0.0016 | 2.63 [1.46, 4.75]  P=0.0013 | 3.98 [1.88, 8.41]  P=0.00031 | 1.82 [0.87, 3.79]  P=0.11 |

Supp Table 3. All BLME thresholds for ROC curve analyses

|  |  |  |  |  |  |
| --- | --- | --- | --- | --- | --- |
| *erf* threshold | 0.7 | 0.8 | 0.9 | 0.99 | 0.999 |
| All Genes | 0.71 [0.57, 0.85]  P=0.022 | 0.73 [0.59, 0.88]  P=0.012 | 0.77 [0.63, 0.91]  P=0.0035 | 0.82 [0.69, 0.95]  P=0.0006 | 0.83 [0.68, 0.97]  P=0.0004 |
| C9*orf72* | 0.58 [0.31, 0.86]  P=0.60 | 0.58 [0.28, 0.88]  P=0.60 | 0.59 [0.27, 0.91]  P=0.57 | 0.64 [0.35, 0.93]  P=0.38 | 0.65 [0.34, 0.95]  P=0.35 |
| *GRN* | 0.84 [0.68, 1.00]  P=0.057 | 0.89 [0.75, 1.00]  P=0.03 | 0.91 [0.79, 1.00]  P=0.022 | 0.95 [0.88, 1.00]  P=0.011 | 0.97 [0.90, 1.00]  P=0.0088 |
| *MAPT* | 0.74 [0.49, 0.99]  P=0.13 | 0.74 [0.49, 0.99]  P=0.13 | 0.80 [0.60, 1.00]  P=0.058 | 0.87 [0.72, 1.00]  P=0.019 | 0.89 [0.77, 1.00]  P=0.014 |

Supp Figure 1. ROC curves for 24-month conversion prediction at all BLME thresholds


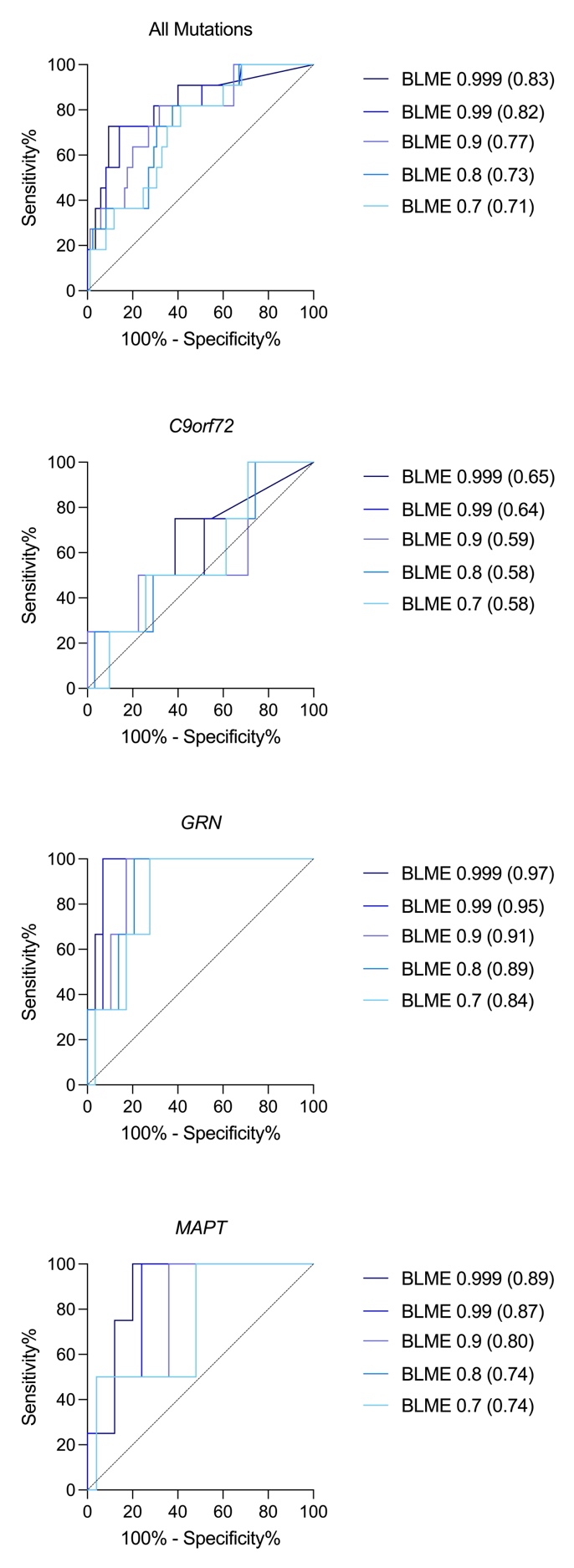


Supp Figure 2. Integration of BLME into translational pathways. Schematic illustrating how longitudinal MRI, cognitive, and biofluid markers in presymptomatic C9orf72, GRN, and MAPT carriers could theoretically be integrated using joint Bayesian predictive distributions to estimate disease proximity and inform participant prioritization and endpoint monitoring for FTLD prevention trials.


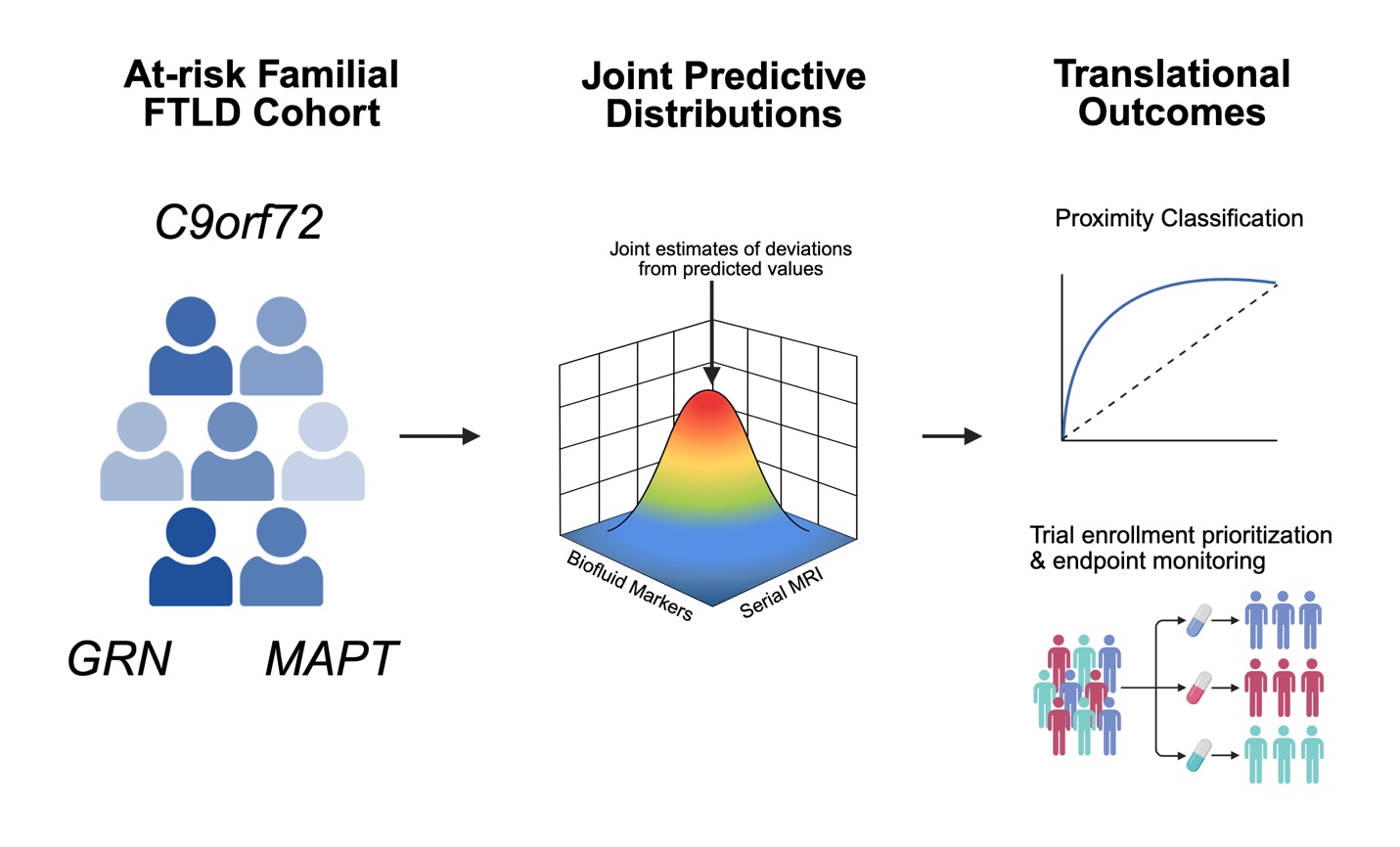


**Acknowledgements**

The authors would like to thank all study participants and their families for their contributions, as well as thank the support staff at each of the participating sites. This study was supported by the ALLFTD Consortium (U19: AG063911, funded by the National Institute on Aging and the National Institute of Neurological Diseases and Stroke) and the former ARTFL and LEFFTDS Consortia (ARTFL: U54 NS092089, funded by the National Institute of Neurological Diseases and Stroke and National Center for Advancing Translational Sciences; LEFFTDS: U01 AG045390, funded by the National Institute on Aging and the National Institute of Neurological Diseases and Stroke). Additional support for this work: J.C.V.S., L.C.J. and H.S. are supported by the Dioraphte Foundation grant 09-02-03-00, Association for Frontotemporal Dementias Research Grant 2009, Netherlands Organization for Scientific Research grant HCMI 056-13-018, ZonMw Memorabel (Deltaplan Dementie, project number 733 051 042), ZonMw Onderzoeksprogramma Dementie (YOD-INCLUDED, project number10510032120002), EU Joint Programme-Neurodegenerative Disease Research-GENFI-PROX, Alzheimer Nederland and the Bluefield Project. R.S-V. is supported by Alzheimer’s Research UK Clinical Research Training Fellowship (ARUK-CRF2017B-2) and has received funding from Fundació Marató de TV3, Spain (grant no. 20143810). C.G. received funding from EU Joint Programme-Neurodegenerative Disease Research-Prefrontals Vetenskapsrådet Dnr 529-2014-7504, EU Joint Programme-Neurodegenerative Disease Research-GENFI-PROX, Vetenskapsrådet 2019-0224, Vetenskapsrådet 2015-02926, Vetenskapsrådet 2018-02754, the Swedish FTD Inititative-Schörling Foundation, Alzheimer Foundation, Brain Foundation, Dementia Foundation and Region Stockholm ALF-project. R.V. has received funding from the Mady Browaeys Fund for Research into Frontotemporal Dementia. J.L. received funding for this work by the Deutsche Forschungsgemeinschaft German Research Foundation under Germany’s Excellence Strategy within the framework of the Munich Cluster for Systems Neurology (EXC 2145 SyNergy—ID 390857198). M.O. has received funding from Germany’s Federal Ministry of Education and Research (BMBF). E.F. has received funding from a Canadian Institute of Health Research grant #327387. M.M. has received funding from a Canadian Institute of Health Research operating grant and the Weston Brain Institute and Ontario Brain Institute. J.B.R. has received funding from the Welcome Trust (103838; 220258) and is supported by the Cambridge University Centre for Frontotemporal Dementia, the Medical Research Council (MC_UU_00030/14; MR/T033371/1) and the National Institute for Health Research Cambridge Biomedical Research Centre (NIHR203312). For the purpose of open access, the author has applied a CC BY public copyright licence to any Author Accepted Manuscript version arising from this submission FM is supported by the Tau Consortium and has received funding from the Carlos III Health Institute (PI19/01637). J.D.R. is supported by the Bluefield Project and the National Institute for Health and Care Research University College London Hospitals Biomedical Research Centre, and has received funding from an MRC Clinician Scientist Fellowship (MR/M008525/1) and a Miriam Marks Brain Research UK Senior Fellowship. Several authors of this publication (J.C.V.S., M.S., R.V., A.d.M., M.O., R.V., J.D.R.) are members of the European Reference Network for Rare Neurological Diseases (ERN-RND) - Project ID No 739510. This work was also supported by the EU Joint Programme—Neurodegenerative Disease Research GENFI-PROX grant [2019-02248; to J.D.R., M.O., B.B., C.G., J.C.V.S. and M.S. B.A. receives research support from the Centers for Disease Control and Prevention, the National Institutes of Health (NIH), Ionis, Alector and the CJD Foundation. He has provided consultation to Acadia, Ionis and Sangamo. E.B. receives research support from the NIH and Lewy Body Dementia Association. B.F.B. has served as an investigator for clinical trials sponsored by Alector, Biogen, Transposon and Cognition Therapeutics. He serves on the Scientific Advisory Board of the Tau Consortium which is funded by the Rainwater Charitable Foundation. He receives research support from NIH. A.L.B. receives research support from the NIH, the Tau Research Consortium, the Association for Frontotemporal Degeneration, Bluefield Project to Cure Frontotemporal Dementia, Corticobasal Degeneration Solutions, the Alzheimer’s Drug Discovery Foundation and the Alzheimer’s Association. He has served as a consultant for Aeovian, AGTC, Alector, Arkuda, Arvinas, Boehringer Ingelheim, Denali, GSK, Life Edit, Humana, Oligomerix, Oscotec, Roche, TrueBinding, Wave, Merck and received research support from Biogen, Eisai and Regeneron. B.C.D. is a consultant for Acadia, Alector, Arkuda, Biogen, Denali, Eisai, Genentech, Lilly, Merck, Novartis, Takeda and Wave Lifesciences; receives royalties from Cambridge University Press, Elsevier and Oxford University Press; and receives grant funding from the NIA, the National Institute of Neurological Disorders and Stroke, the National Institute of Mental Health and the Bluefield Foundation. K.D.-R. receives research support from the NIH and serves as an investigator for a clinical trial sponsored by Lawson Health Research Institute. L.F. receives research support from the NIH. N.G. has participated or is currently participating in clinical trials of anti-dementia drugs sponsored by Bristol Myers Squibb, Eli Lilly/Avid Radiopharmaceuticals, Janssen Immunotherapy, Novartis, Pfizer, Wyeth, SNIFF (The Study of Nasal Insulin to Fight Forgetfulness) and the A4 (The Anti-Amyloid Treatment in Asymptomatic Alzheimer’s Disease) trial. She receives research support from Tau Consortium and the Association for Frontotemporal Dementia and is funded by the NIH. N.G.-R. receives royalties from UpToDate and has participated in multicenter therapy studies by sponsored by Biogen, TauRx, and Lilly. He receives research support from the NIH. M.G. receives grant support from the NIH, Avid and Piramal; participates in clinical trials sponsored by Biogen, TauRx and Alector; serves as a consultant to Bracco and UCB; and serves on the editorial board of Neurology. Site PI or SubI for several industry (Alector, Janssen, Biogen, Cogito Tx) sponsored clinical trials with funding through Emory Office of Sponsored Programs. L.H. receives Research Funding from Abbvie, Acumen, Alector, Biogen, BMS, Eisai, Genentech/Roche, Janssen/J&J, Transposon, UCB, Vaccinex. Consulting fees from Biogen, Cortexyme, Eisai, Medscape, Prevail/Lilly. G.-Y.H. has served as an investigator for clinical trials sponsored by AstraZeneca, Eli Lilly and Roche/Genentech. He receives research support from the Canadian Institutes of Health Research and the Alzheimer Society of British Columbia. E.D.H. receives research support from the NIH D.I. receives support from the NIH, BrightFocus Foundation and Penn Institute on Aging. D. Knopman serves on the data and safety monitoring board of the DIAN-TU study; is a site principal investigator for clinical trials sponsored by Biogen, Lilly and the University of Southern California; and is funded by the NIH. I.L.'s research is supported by the National Institutes of Health grants: 2R01AG038791-06A, U01NS100610, U01NS80818, R25NS098999; U19 AG063911-1 and 1R21NS114764-01A1; the Michael J Fox Foundation, Parkinson Foundation, Lewy Body Association, CurePSP, Roche, Abbvie, Biogen, Centogene. EIP-Pharma, Biohaven Pharmaceuticals, Novartis, Brain Neurotherapy Bio and United Biopharma SRL - UCB. She is a Scientific advisor for Amydis and Rossy Center for Progressive Supranuclear Palsy University of Toronto . She receives her salary from the University of California San Diego and as Chief Editor of Frontiers in Neurology. I.R.M. receives research funding from the Canadian Institutes of Health Research, the Alzheimer’s Association US, the NIH and the Weston Brain Institute. M.F.M. receives research support from the NIH. C.U.O. receives research funding from the NIH, Lawton Health Research Institute, National Ataxia Foundation, Alector and Transposon. He is also supported by the Robert and Nancy Hall Brain Research Fund, the Jane Tanger Black Fund for Young-Onset Dementias and a gift from Joseph Trovato. He is a consultant with Alector Inc., Acadia Pharmaceuticals, and Reata Pharmaceuticals. R.R. receives research funding from the NIH and the Bluefield Project to Cure Frontotemporal Dementia. R.R. is on the scientific advisory board of Arkuda Therapeutics and receives royalties from progranulin-related patent. She is also on the scientific advisory board of the Fondation Alzheimer. E.M.R. receives research support from the NIH. E.D.R. has received research support from the NIH, the Bluefield Project to Cure Frontotemporal Dementia, the Alzheimer's Association, the Alzheimer's Drug Discovery Foundation, the BrightFocus Foundation, and Alector; has served as a consultant for AGTC and on a data monitoring committee for Lilly; and owns intellectual property related to tau and progranulin. H.J.R. has received research support from Biogen Pharmaceuticals, has consulting agreements with Wave Neuroscience, Ionis Pharmaceuticals, Eisai Pharmaceuticals, and Genentech, and receives research support from the NIH and the state of California. M.C.T. has served as an investigator for clinical trials sponsored by Biogen, Avanex, Green Valley, Roche/Genentech, Bristol Myers Squibb, Eli Lilly/Avid Radiopharmaceuticals and Janssen. She receives research support from the Canadian Institutes of Health Research.
